# Analgesic Effect of Odontopaste and a Compound Intracanal Medicament Between Root Canal Therapy Appointments

**DOI:** 10.17795/jjnpp-12473

**Published:** 2013-11-01

**Authors:** Behrooz Eftekhar, Eskandar Moghimipour, Pejman Pourakbar Jahandideh, Sahar Jalali, Mahsa Mahmoudian

**Affiliations:** 1 Endodontic Department, School of Dentistry, Ahvaz Jundishapur University of Medical Sciences, Ahvaz, IR Iran; 2 Cellular and Molecular Research Center, Ahvaz Jundishapur University of Medical Sciences, Ahvaz, IR Iran

**Keywords:** Anti-Inflammatory Agent, Clindamycin, Periapical Periodontitis, Root Canal Irrigants, Triamcinolone Acetonide

## Abstract

**Background:**

Pain experience makes a serious anxiety for both patient and clinician before and after root canal treatment. Pain is a complex psychophysiologic phenomenon.

**Objectives:**

The aim of this randomized control trial study was to evaluate the analgesic effect of Odontopaste® and a corticosteroid containing compound medicament between root canal therapy appointments.

**Materials and Methods:**

One hundred and twenty lower first and second mandibular molars with spontaneous pain and sensitivity to percussion were selected and divided into three groups (40 patients per each group). After root canal preparation, patients were entered one of these groups randomly. Root canals in group 1 were dressed with Odontopaste, in group 2 with a compound intracanal medicament, and in group 3 with placebo. Patients determined their pain rate and percussion sensitivity on Heft-parker VAS diagram, before the first appointment and 24 hours and 7 days after that.

**Results:**

Spontaneous pain and Percussion sensitivity score averages of 24 hours after the first appointment in group 1 and group 2 were less than group 3, which indicates statistically significant difference between these groups. There was no statistically significant difference between these groups after 7 days neither on spontaneous pain nor percussion sensitivity.

**Conclusions:**

Odontopaste® and compound intracanal medicaments resulted in statistically significant reduction in postoperative pain and percussion sensitivity after 24 hours, but there was no statistically significant difference after 7 days with placebo.

## 1. Background

Pain experience makes a serious anxiety for both patient and clinician before and after root canal treatment. Pain is a complex psychophysiologic phenomenon (1). Inflammation can affect pain threshold by inflammation mediators which alter intrapulpal local pressure, lead to nociceptor proliferation, and reduce stimulation threshold (2, 3). Agreement exists that apical periodontitis in necrotic teeth is more resistant to heal than vital teeth after endodontic treatment and may require multiple appointments (4). Moreover in cases with apical destruction during preparation of canal, there is more severe pain experience between and after root canal treatment appointments. Pain is a subjective phenomenon and its measurement can be difficult and is affected by individual experiences (4). Several factors influence pain during and after root canal therapy such as gender, age, tooth position, intracanal irrigants, intracanal dressings, root canal fillings, number of appointments, and apical periodontitis (5, 6).

Maintaining of patency during root canal cleaning and shaping, directly irritates periapical tissue and inadvertently extrudes bacteria, its byproducts, necrotic debris, and irritant solutions beyond the apex (7, 8). Therefore use of intracanal medicaments can kill residual bacteria and may reduce periradicular inflammation and obviate intracanal exudation (9). One of these intracanal medicaments is Odontopaste which is a zinc oxide-based root canal paste with clindamycin hydrochloride 5% and triamcinolone acetonide 1%, formulated and developed in Australia (Australian Dental Manufacturing, Kenmore Hills, Qld, Australia). This antibiotic provides a bacteriostatic activity in addition to benefits of a zinc oxide paste. It acts like a temporarily dressing material and prevents bacterial repopulation both within the root canal and the paste itself. On the other hand, the steroid part, triamcinolone acetonide, temporarily reduces inflammation. It is useful for transient reduction of postoperative pain. Odontopaste is not designed to eliminate all of the bacteria within the root canal, therefore it is important to continue using the standard protocols of root canal preparation for example rinsing by Sodium hypochlorite following Ethylenediaminetetraacetic acid (EDTA) (10). Mixing of additional calcium hydroxide in a 50:50 combination with Odontopaste is not recommended because the steroid component destroys immediately by increased alkalinity, and it offers only minimal benefits over the use of calcium hydroxide alone. Odontopaste contains calcium hydroxide but only at a 0.5% level which has been proven to be optimal for the preservation of the steroid component (10-12). Odontopaste does not stain teeth, hence, no bleaching is required following the use of Odontopaste (12). Odontopaste is available in an 8 gram tube (10).

## 2. Objectives

The aim of this randomized control trial study was to evaluate the analgesic effect of Odontopaste® and a corticosteroid containing compound medicament between root canal therapy appointments.

## 3. Materials and Methods

This randomized control trial study was performed on 120 patients (18-35 years old) at the endodontic department of Ahvaz dental school from December 2011 to October 2012. Sample size was calculated after a pilot study with 30 patients by using statistical software (SPSS version 16).

### 3.1. Ethical Considerations

The outline of this clinical control trial was approved by the Ethics in Clinical Research Committee of Ahvaz University of Medical Sciences.

### 3.2. Case Selection

The inclusion criteria were:

- Selected patients should have just one lower posterior first or second molar with spontaneous pain or sensitivity to percussion before the treatment.

- Patients should not have history of any oral disease, sensitivity to antibiotic or corticosteroid, and should be without any systemic problems.

- Female patients should not be pregnant or lactescent.

- Selected teeth should be completely formed without any root resorption and pathological problems such as periapical lesions.

- The patients should not have used NSAIDs at least 3 hours before the treatment.

The exclusion criteria were:

- Any procedural accident during the treatment.

- Use of analgesics by the patient between appointments.

- Those patients who could not be followed after seven days or had not been satisfied to continue the study or the pain rate was not recorded at 24 hours after the first appointment.

- Any time after the first appointment, those cases who had severe pain, would be given analgesics and excluded from the study.

**Figure 1.**
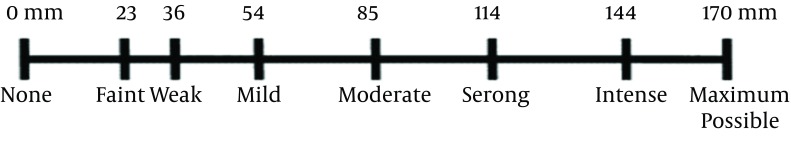
Heft-Parker VAS Used for the Assessment of Pain. The Millimeter Demarcation (Numbers) Was Not Shown on the VAS Used by Patients.

Total 120 patients were selected and divided into three groups randomly by random number table. Canals were dressed with: Odontopaste medicament (Group 1), a compound medicament containing triamcinolone acetonide and clindamycin hydrochloride (Group 2), and placebo (Group 3). The procedural risks and benefits of treatment were explained to each patient and a written consent was taken before endodontic treatment. English Heft-parker diagram (Figure 1) was translated to Persian by a person who was fluent in both languages (13). A common guidance form was given to all patients.

Patients determined their spontaneous pain and percussion sensitivity rates on the Heft-parker VAS diagram, in the beginning of any appointment. Complete anesthesia was achieved by local anesthetic solution (1:80,000 lidocaine, persocaine, Darupaksh, Iran). Access preparation was performed after rubber dam isolation by using a high speed hand piece with size 02 tapered fissure diamond bur. Coronal half of canals were prepared by No. 4, 3, 2 Gates-Glidden burs with crown down technique. After pulpectomy, working length (WL) was determined by digital radiography using ISO K-file #15 (Mani INC, Kiyohara Industrial Park, Utsunomiya, Touchigi, Japan). Optimal length has been 1 to 2 mm short of the apex, and then canals were cleaned and shaped according to Protaper® (Dentsply Maillefer, Bal- laigues, Switzerland) manufacturer orders. During canal preparation with crown-down technique by Gates Glidden and rotary Ni-Ti files, apical canal patency was maintained. After each file, root canals were irrigated with 5.25% sodium hypochlorite (NaOCl), and then 17% EDTA, finally canals were irrigated with normal saline. Canals were dried by absorbent paper points and the intracanal medicament of each group was inserted to each canal by a spiral lentulo. Finally a #25 K-file was inserted up to working length to confirm complete root canal filing with the medicament. The practitioner was blind about intracanal medicament and the assistant gave appropriate medicament on the glass pad, according to random number table. To avoid recontamination of the canal, access cavity was loosely packed with a sterile cotton pellet and was covered by Cavisol (Impermanent Filling Material, Golchie Co, Iran) between appointments. The patients were recalled to record the intensity of spontaneous pain and percussion sensitivity, on the Heft-parker VAS diagram, 24 hours after the first appointment. In the beginning of the 7-day appointment, patients determined their pain rates on the VAS diagram. The canals were obturated by gutta-percha with lateral condensation up to working length. The assistant also recorded a code for each medicament (Q-R-S) on patient file. Data was given to statistician blindly by codes, so the patient, practitioner and statistician were blind, and this study was triple-blind. If the patient required analgesics at any time, it would be prescribed and he or she would be excluded from the study. The placebo contained: Gel form Hydroxyl ethyl cellulose 6%, methyl paraben 0.2%, Sodium metabisulphide 0.2%, Sodium sulfate 1%, and Zinc oxide 2%.

## 4. Results

All of the 120 patients in this study had acute apical periodontitis and spontaneous pain. Patients were equally divided into three groups. In group 1, patients were treated with Odontopaste®, in group 2 patients were treated with a compound intracanal medicament containing corticosteroid, and patients in group 3 received placebo. Overall spontaneous pain score average of all patients in the three groups before the treatment was 128.8 ± 18.71 which decreased to 33.2 ± 11.31and 1.6 ± 3.87, 24 hours and 7 days after the first appointment respectively (Table 1). Also, overall percussion sensitivity score average of patients of these groups before the treatment was 134 ± 16.15, which reduced to 36.8 ± 11.51 and 12.6 ± 3.77, 24 hours and 7 days after the first appointment respectively (Table 2). After Homogeneity of Variances test, results of each group in 3 time intervals were analyzed by ANOVA. Spontaneous pain score average of 24 hours after the first appointment was 29.3 ± 11.9 in group 1, 30.1 ± 12.1 in group 2, and 40.1 ± 13.09 in group 3 (Table 3). Although there was a significant difference between both medicaments (group 1, 2) and placebo (group 3) (P = 0.00, 0.001), the difference between the results of groups 1 and 2 was not significant (P = 0.958).

**Table 1.** Overall Spontaneous Pain Score Average for All of Patients in Different Periods of Time

Spontaneous Pain	Means	Deviation	95% Confidence Interval	
			Lower Bound	Upper Bound
**Before treatment**	128.883	18.71	125.178	132.589
**After treatment**				
24 hours	33.200	11.31	30.961	35.439
7 days	1.650	3.87	0.883	2.417

**Table 2.** Overall Percussion Sensitivity Score Average for All of Patients in Different Periods of Time

Percussion Sensitivity	Means	Deviation	95% Confidence Interval	
			Lower Bound	Upper Bound
**Before treatment**	134.000	16.15	130.801	137.199
**After treatment**				
24 hours	36.867	11.51	34.587	39.147
7 days	12.625	3.77	11.878	13.372

**Table 3.** Descriptive Analysis of Spontaneous Pain Scores

Spontaneous Pain	Numbers	Means	Deviation	Minimum	Maximum
**Before treatment**					
1	40	128.3	20.24388	85.00	160.00
2	40	129.4	20.11097	90.00	160.00
3	40	128.9	21.11797	85.00	165.00
Total	120	128.8	20.32765	85.00	165.00
**24 hours after treatment**					
1	40	29.3	11.92796	10.00	56.00
2	40	30.1	12.10382	10.00	56.00
3	40	40.1	13.09117	20.00	70.00
Total	120	33.2	13.24533	10.00	70.00

Percussion sensitivity score average of 24 hours after the first appointment in groups 1 to 3 were 32.42 ± 12.44, 33.80 ± 12.18, and 44.37 ± 13.17, respectively. Although there was a significant difference between both medicaments (group 1, 2) and placebo (group 3) (P = 0.00, P = 0.001), the difference between the results of groups 1 and 2 was not significant (P = 0.877) (Table 4). For pair-wise evaluation of spontaneous pain of 24 hours after the first appointment between the groups, Tukey HSD test was used. According to this test, the difference between average spontaneous pain in groups 1 and 2 was 0.7 (not significant P = 0.958), in groups 1 and 3 was 10.85 (P = 0.00), and in groups 2 and 3 was 10.07 (P = 0.001) (Table 5). For pair-wise evaluation of percussion sensitivity of 24 hours after the first appointment between the groups, Tukey HSD test was used. According to this test, the difference between average spontaneous pain in groups 1 and 2 was 1.3 (not significant P = 0.877), in groups 1 and 3 was 11.9 (P = 0.00), and in groups 2 and 3 was 10.5 (P = 0.001) (Table 6).

**Table 4.** Descriptive Analysis of Percussion Sensitivity Scores

percussion Sensitivity	Numbers	Means	Deviation	Minimum	Maximum
**Before treatment**					
1	40	133.8	17.78100	98.00	164.00
2	40	134.2	18.03586	100.00	165.00
3	40	134	17.25228	100.00	165.00
Total	120	134	17.54418	98.00	165.00
**24 hours after treatment**					
1	40	32.42	12.44864	12.00	61.00
2	40	33.80	12.18700	12.00	60.00
3	40	44.37	13.17668	25.00	75.00
Total	120	36.86	13.60544	12.00	75.00
**7 days after treatment**					
1	40	11.75	3.33397	5.00	19.00
2	40	12.87	3.35267	5.00	20.00
3	40	13.25	5.37683	5.00	23.00
Total	120	12.62	4.14863	5.00	23.00

**Table 5.** The Spontaneous Pain Pair-Wise Comparison, 24 Hours After the First Appointment Between the Groups on Tukey HSD Test

Group	Mean Difference	SE	Significant	95% Confidence Interval	
				Lower Bound	Upper Bound
**1 & 2**	0.7	2.76935	0.958	-5.7992	7.3492
**1 & 3**	-10.85	2.76935	0.000	-17.4242	-4.2758
**2 & 1**	0.7	2.76935	0.958	-7.3492	5.7992
**2 & 3**	-10.07	2.76935	0.001	-16.6492	-3.5008
**3 & 1**	10.85	2.76935	0.000	4.2758	17.4242
**3 & 2**	10.07	2.76935	0.001	3.5008	16.6492

**Table 6.** The Percussion Sensitivity Pair-Wise Comparison, 24 Hours After the First Appointment Between the Groups Based on Tukey HSD Test

Group	Mean Difference	SE	Significant	95% Confidence Interval	
				Lower Bound	Upper Bound
**1 & 2**	1.37500	2.81992	0.877	-5.3192	8.0692
**1 & 3**	-11.95000	2.81992	0.000	-18.6442	-5.2558
**2 & 1**	-1.37500	2.81992	0.877	-8.0692	5.3192
**2 & 3**	-10.57500	2.81992	0.001	-17.2692	-3.8808
**3 & 1**	11.95000	2.81992	0.000	5.2558	18.6442
**3 & 2**	10.57500	2.81992	0.001	3.8808	17.2692

Patients with Odontopaste® and compound intracanal medicament experienced significantly less spontaneous pain and percussion sensitivity than placebo group (P = 0.00) after 24 hours from the first appointment. Welch analysis could not find any significant difference between percussion sensitivity score average of groups, 7 days after the first appointment (P = 0.203). The average score of spontaneous pain in groups 1 and 2 was 0 after 7 days, so it was not possible to compare groups at this period of time.

## 5. Discussion

The most significant finding of this study was reduction in spontaneous pain and percussion sensitivity by use of corticosteroid containing material as an interappointment medicament compared to control group. Endodontic literature contains few studies regarding the efficacy of Ledermix (Haupt Pharma GmbH, Wolfratshausen, Germany) a corticosteroid/antibiotic mixture in comparison with other intracanal medicaments, but there is a few studies about Odontopaste. Ehrmann et al. found that the flare-up (pain and/or swelling) scores mean of Ledermix was significantly lower than the flare-up scores mean of control group (14). Our study showed similar pain and percussion sensitivity reduction for Odontopaste® in acute apical periodontitis, 24 hours after the first appointment. One of the active ingredients of Odontopaste® is triamcinolone. Abbott et al. in their in-vitro study evaluated the rapidity and prompt action of triamcinolone in Ledermix. They concluded that the rate of release of triamcinolone is in maximum level during the first 3-8 hours, but it reduced after 8 hours (15). There is controversy about antimicrobial efficacy of intracanal medicaments, some authors believe that these are not efficient enough against microbial biofilm, and have mentioned that antimicrobial effect is necessary (16). Trope did not found any difference between Ledermix, Calcium Hydroxide and Formocresol in flare-up rate reduction. He reported very low rate of flare-up (only 2.53%: 12 in 474 cases) which may be due to his meticulous adherence to exclusion criteria (17). Genet et al. observed a flare-up rate of 27% in all treated cases. These investigators also found a direct correlation between preoperative pain and the incidence of postoperative pain (18). Seltzer criticized the use of corticosteroids in endodontic therapy by stating that the disadvantage of using corticosteroids derives from its effects on inflammatory cells. He stated that inflammatory cells hamper the process of phagocytosis and protein syntheses, which may result in delayed healing (19). But Abbott disproved these findings and said that even if corticosteroids cause some adverse effects, they are of minor degree and insignificant (20). Ledermix Paste has been recommended for endodontic use due to its anti-inflammatory effect (21). Odontopaste has recently been released onto the dental market, similarly due to its anti-inflammatory effect and no adverse effect on tooth color. The main difference between Odontopaste and Ledermix Paste is in its antibiotic ingredient: clindamycin hydrochloride in Odontopaste replaces demeclocycline hydrochloride in Ledermix Paste. Clindamycin hydrochloride has an equivalent spectrum of antibacterial activity but exhibits minimal staining of teeth (22). There are different recommendations for mixing Ledermix or Odontopaste with calcium hydroxide. Calcium hydroxide can be mixed with the Ledermix paste (as an approximate 50:50 mixture) or it can be used as a separate subsequent dressing in canal (15, 23). Although Odontopaste itself has about 0.5% calcium hydroxide in its combination, mixing of additional calcium hydroxide in a 50:50 combination with Odontopaste is not recommended. Athanassiadis et al. observed rapid loss of steroid when Odontopaste or Ledermix was mixed with calcium hydroxide powder, although the overall destruction of steroid in Odontopaste was less (11).

It seems that there is no real advantage for mixing calcium hydroxide with Ledermix or Odontopaste. There is no significant antibacterial enhancement in these calcium hydroxide combinations when compared to calcium hydroxide alone (24). So in this study calcium hydroxide was not used with Odontopaste or the other medicament. There are controversies about the effect of age on postoperative pain incidence. Balban found that with advancing age, the percentage of cases with acute exacerbation of interappointment pain decreased. The reason he gave was that, in old age the pulp canal size decreases significantly. Thus there would be a decreased volume of debris, reduced flow of blood to the alveolus, and thus reduced inflammatory response to infection (25). Another study, a prospective randomized trial conducted by Morse et al. concluded that no direct or indirect association could be established between the incidence of interappointment pain and age of patients (26). On the contrary, there are some studies that reject the idea given above. Some researchers concluded that patients above 50 years are more prone to develop interappointment flare-ups. The reason given was that, the cementum deposition increases with age; therefore, coronal transportation of radiographic apex may occur. This would lead to increased possibility of developing errors in determining the working length and apical extrusion of the debris, thus patients in old age have more chance of developing interappointment flare-up than younger patients (25, 27). Due to these controversies, one of the inclusion criteria in this study was age limitation.

Corticosteroids containing intracanal medicaments like Odontopaste® reduce postoperative spontaneous pain rate and percussion sensitivity significantly, 24 hours after the first appointment, but our study did not show any significant effect 7 days after the first appointment.
